# Alternativas diagnósticas y de tratamiento para la corrección de asimetrías faciales: revisión bibliográfica

**DOI:** 10.21142/2523-2754-1001-2022-098

**Published:** 2022-03-30

**Authors:** Braulio Rafael Rojas Reyna, María Isabel Ramírez Prado, Norma Idalia Orozco Orozco, Luis Renán Rodríguez Pérez, Ana Alicia Simg Alor, Víctor Manuel Quirarte Echavarría

**Affiliations:** 1 Facultad de Odontologia de la Universidad Veracruzana Campus Minatitlan. Veracruz, Mexico. brrojas0498@gmail.com, isramirez@uv.mx, norozco@uv.mx, luisrodriguez@uv.mx, asimg@uv.mx, vquirarte@uv.mx Universidad Veracruzana Facultad de Odontologia Universidad Veracruzana Campus Minatitlan Veracruz Mexico brrojas0498@gmail.com isramirez@uv.mx norozco@uv.mx luisrodriguez@uv.mx asimg@uv.mx vquirarte@uv.mx

**Keywords:** asimetría facial, deformidad dentofacial, tomografía computarizada, cirugía ortognática, facial asymmetry, dentofacial deformity, computed tomography, orthognathic surgery

## Abstract

La simetría facial hace referencia a una coincidencia completa de cada componente facial sobre el plano sagital, mientras que la asimetría se refiere a la diferencia bilateral entre dichos componentes. La presencia de una simetría bilateral perfecta casi nunca se presenta en el cuerpo humano, es decir, no se encuentra con facilidad, por lo que es más común que en los individuos predomine la asimetría facial. Sin embargo, este padecimiento puede derivarse de problemas funcionales y estéticos. Mediante esta investigación, se busca determinar las alternativas, el diagnóstico y plan de tratamiento ideal para la corrección de cada tipo de asimetría facial de la mano de la cirugía ortognática. Para ello, se realizó la revisión de artículos publicados desde el 2015 hasta la fecha. Se seleccionó aquellos que estuvieran enfocados en describir o evaluar la simetría y asimetría tanto facial como dentofacial, el tipo de diagnóstico o el plan de tratamiento, incluyendo casos clínicos. Se utilizó el buscador de Google, SciELO y bases de datos de interés médico, como PubMed, PMC y Medigraphic. De acuerdo con las investigaciones y tratamientos realizados durante los últimos años dentro de la clínica dental, queda comprobado que el tratamiento ortognático, acompañado de una planificación previa, es la mejor opción para el manejo de las asimetrías faciales esqueletales.

## Abstract (en)

Facial symmetry refers to a complete coincidence of each facial component on the sagittal plane, while asymmetry refers to the bilateral difference between these components. The presence of perfect bilateral symmetry is almost never present in human, so it is more common for individuals to predominate facial asymmetry. However, this condition can result from functional and esthetic problems. This research seeks to determine the alternatives, diagnosis and ideal treatment plan for the correction of each type of facial asymmetry through orthognathic surgery. For this purpose, a review of articles published between 2015 to date was carried out. Selecting those that were focused on describing or evaluating facial and dentofacial symmetry and asymmetry, type of diagnosis or treatment plan, including clinical cases. The Google search engine, SciELO and databases of medical interest, such as PubMed, PMC and Medigraphic were used. According to the research and treatments carried out during the last few years in the dental clinic, it has been proven that orthognathic treatment accompanied by previous planning is the best option for the management of skeletal facial asymmetries.

## INTRODUCCIÓN

Los rasgos faciales y dentales son una característica única y diferente en cada persona. Sin la necesidad de ser un especialista en el tema, es posible observar las diferencias faciales entre seres humanos, a veces, de acuerdo con la etnia, la nacionalidad o la rama familiar. Es posible pensar que existen similitudes en la forma de la cara de cierto grupo de personas; sin embargo, la cara comúnmente suele presentarse con un grado leve de asimetría. Esta ligera asimetría, también conocida como simetría relativa, asimetría subclínica o asimetría normal, suele pasar desapercibida por sus portadores cuando es menor de 3 mm con respecto a puntos prominentes, como la comisura oral o el nivel de la frente ^1^^-^^3^. 

El cuerpo humano tiende a presentar un desarrollo esquelético simétrico bilateral. La simetría facial o dentofacial es una ocurrencia común en la naturaleza y consiste en la coincidencia o igualdad completa sobre lados opuestos en cuanto a tamaño, ubicación de estructuras óseas, formas y disposición de cada componente facial sobre el plano sagital ^4^^-^^7^, con cuya suma se obtiene el balance del complejo dentofacial ^8^. Por el contrario, las asimetrías implican alteraciones de tamaño, forma o malposición de estructuras faciales ^9^. La simetría bilateral perfecta no se encuentra con facilidad y casi nunca existe en el cuerpo humano, por lo cual es normal presentar cierto grado de asimetría en las estructuras faciales ^10^, como resultado de causas congénitas, ambientales y factores funcionales de origen bucodental. Existen asimetrías de una sola causa que presentan varios patrones según las características de la persona, el momento en el que aparecen, la compensación muscular y el desarrollo musculoesquelético de la cara. En consecuencia, clasificar a la asimetría facial se considera difícil ^11^. 

La asimetría de tipo esqueletal (clase III) es considerada uno de los problemas más difíciles de corregir con ortodoncia. Puede ocurrir un cambio funcional de la mandíbula en pacientes en crecimiento que acompaña a una clase III, debido a la constricción del arco maxilar y las interferencias oclusales ^12^. Es particularmente común en individuos con ancestros asiáticos (en la población china, se encuentra en un 12%) y menor su aparición en europeos (1,5 a 5,3%) y norteamericanos caucásicos (1 a 4%) ^13^.

La presente revisión bibliográfica se realiza con la finalidad de informar al lector desde qué punto se puede considerar a una asimetría como un rasgo normal o anormal, así como determinar los métodos de evaluación, los tipos de diagnóstico y el plan de tratamiento ideal para su corrección, de acuerdo con las características y etiología de las diferentes asimetrías que pueden presentarse en la cara y dentro de la boca.

## MATERIALES Y MÉTODOS

La información reunida para la presente búsqueda bibliográfica fue recabada de artículos desde 2015 hasta la fecha. Se seleccionó aquellos que estuvieran enfocados en describir o evaluar la simetría y asimetría tanto facial como dentofacial, así como algunos factores de estas, tipos de diagnóstico o planes de tratamiento. 

Las palabras utilizadas para encontrar los artículos fueron “Simetría facial”, “Asimetría facial”, “Asimetría dentofacial”, “Evaluación de asimetrías faciales”, “Manejo de asimetrías faciales”, tanto en español como en inglés.

Se utilizó el buscador de Google, SciELO y bases de datos de interés médico, como PubMed, PMC y Medigraphic. De todas estas, se reunieron 53 artículos publicados entre los años establecidos y, una vez revisada la información, se descartaron algunos y se seleccionó solo 39, de acuerdo a los criterios establecidos.

## PRESENCIA DE ASIMETRÍA EN EL ROSTRO HUMANO

La asimetría se encuentra en pacientes con o sin alteraciones cosméticas faciales, quienes llegan a manifestar problemas esqueléticos subyacentes y algunos solo una asimetría facial limitada de tejidos blandos ^14^.

Las asimetrías faciales pueden involucrar al tercio medio e inferior. Autores como Severt y Proffit reportan que la asimetría facial que involucra el tercio superior de la cara equivale solo a un 5% de pacientes; en el tercio medio, solo el 36%; y el 75%, en el tercio inferior, con una desviación lateral del mentón ^15^^,^^16^.

La prevalencia de estas alteraciones faciales puede presentarse con mayor frecuencia en ciertas poblaciones o grupos étnicos; del mismo modo, puede ser mayor en un país que en otro (Tabla 1).

**Tabla 1:** Prevalencia de la asimetría facial en pacientes de ortodoncia en diferentes países, según diversos autores

Autor	País	Prevalencia
Fundas, Bailey *et al*. ^2^	Estados Unidos	12%-37%
Sotoyomar *et al*. ^9^	China	25%
Willems *et al*. ^2^	Bélgica	23%
Samman *et al*. ^2^	Hong Kong	21%
Boeck *et al*. ^2^	Brasil	32%

Elaboración propia.

Entre el número de personas que llegan a presentar una asimetría facial, se pueden identificar asimetrías con ciertas características y con una etiología distinta, siendo más común unas que otras (Tabla 2).

**Tabla 2:** Prevalencia de los tipos de asimetrías faciales más comunes, según diversos autores

Autor	Tipo de asimetría	Prevalencia
Cueto S *et al*. ^17^	Dentofacial	12%
Thiesen *et al*. ^6^	Mandibular	34%
Olate *et al*. ^18^	Por hiperplasia condilar	30%
Cazenave L, Leiva N, Morovic CG ^19^	Por microsomía hemifacial	16%

Elaboración propia.

La mordida de un individuo puede presentar falta de armonía, la cual puede ser considerada como asimétrica. En este sentido, la asimetría facial se puede observar en la línea media dental superior, al encontrarse una desviación lateral de la LMDS con respecto a la línea media facial. Es percibida como desagradable o estéticamente no aceptable ^9^.

En ocasiones, las maloclusiones pueden ser un factor para estas asimetrías en boca y llegan a reflejarse en la cara. 

Según Haraguchi *et al*., cualquier punto de referencia que se desvíe más de 2 mm de la línea media facial es asimétrico. Severt y Proffit encontraron un mayor porcentaje de desviación del mentón de al menos 2 mm desde la línea media en la deformidad de clase III ^20^.

En algunos casos, la desviación del mentón, puede llegar a describirse como asimetría mandibular, que se observa como una diferencia de tamaño, forma y volumen del lado izquierdo y el derecho de la mandíbula, con el mentón como la única estructura desviada, lo cual puede ser la causa de problemas estéticos y funcionales ^15^^,^^21^.

En otro tipo de ocasiones, la desviación del mentón y la mandíbula puede verse influenciada por una maloclusión clase III o por un crecimiento aún más excesivo de uno de los cóndilos (hiperplasia condilar).

### Asimetría por hiperplasia condilar

Se caracteriza por el excesivo desarrollo de la cabeza y del cuello de los procesos condilares, así como de la mandíbula. Puede ser unilateral o bilateral, y estar acompañada de dolor, alteración de la oclusión y disfunción articular con implicaciones estéticas y funcionales, lo cual puede condicionar a una discrepancia dentofacial y asimetría facial ^22^^,^^16^.

La hiperplasia condilar presenta una gran prevalencia entre las asimetrías; sin embargo, esta también puede presentarse con un mayor incremento vertical que horizontal o más sobre un cóndilo en comparación con el otro (Tabla 3).

**Tabla 3:** Prevalencia de la hiperplasia condilar por diversos autores

Autor	Año	Prevalencia
Nitzan ^18^	2008	53% con incrementos de hemimandíbula horizontal
		31% con incrementos de hemimandíbula vertical
		16% con incrementos de hemimandíbula horizontal y vertical
Raijmakers ^18^	2009	64% más común en mujeres, sin una edad específica
Olate *et al*. ^18^	2012	30% en pacientes con asimetría facial
López *et al*. ^16^	2017	57% en el cóndilo del derecho
		43% en el cóndilo izquierdo

Elaboración propia.

La asimetría facial puede ser causada por hiperplasia condilar unilateral. Según la velocidad de desarrollo de este, puede provocar canteamiento oclusal o presencia de mordida abierta posterior, con alteraciones en línea media dental ^18^^,^^23^.

Las desviaciones en el crecimiento del cóndilo mandibular pueden afectar tanto la oclusión funcional como la apariencia de la estética facial. Las razones de estas desviaciones del crecimiento son numerosas y, a menudo, implican un mal funcionamiento a nivel celular ^24^.

### Asimetría por microsomía hemifacial

La microsomía hemifacial presenta un subdesarrollo en la mitad inferior de un lado de la cara ^10^. Los hallazgos característicos incluyen asimetría facial leve, moderada y severa, como resultado de una hipoplasia unilateral maxilar o mandibular; pero su característica clínica principal es la asimetría facial, particularmente de la mandíbula ^19^.

Típicamente, se presentan anomalías como crestas supraorbitales subdesarrolladas, pendiente negativa de las fisuras palpebrales, hipoplasia del hueso malar, así como de las ramas mandibulares y los cóndilos, lo que da como resultado una asimetría facial progresiva ^19^^,^^24^.

### Etiología de las asimetría facial y de sus factores causales

Se ha planteado la hipótesis de que la asimetría, especialmente la fluctuante, está estrechamente relacionada con la inestabilidad del desarrollo, y muchos estudios la han interpretado como un marcador de estrés ambiental durante el desarrollo ^25^.

En muchos pacientes, la asimetría es el resultado de una serie de cambios dentofaciales y puede dar lugar a compensaciones posturales que dificultan la correcta caracterización de esta desarmonía ^2^.

Etiología de las asimetrías dentofaciales (deformidades dentofaciales)

Provienen de causas congénitas musculoesqueléticas que pueden ocasionar alteraciones en la estética y la funcionalidad facial, y por ende en la vida social, lo que condiciona de forma dramática la actividad diaria de quienes la padecen ^17^.

### Etiología de la maloclusión clase III

Los factores etiológicos de las maloclusiones de clase III incluyen un amplio espectro de componentes de compensación esquelética y dental. La afección puede caracterizarse por prognatismo mandibular, retrognatismo maxilar, dentición mandibular retrusiva, dentición maxilar protrusiva y una combinación de los anteriores ^26^.

### Etiología de la asimetría mandibular

• Causa funcional. Suele deberse a una malposición o desplazamiento lateral, cuya causa puede ser oclusal (contactos prematuros, mordidas cruzadas unilaterales), articular o muscular. En estos casos, el desplazamiento es originado por una posición habitual de desviación lateral.

• Causa esqueletal. Es ocasionada por un crecimiento desigual del cóndilo, que se manifiesta en una diferente longitud de las ramas, con la línea media mandibular desplazada hacia el lado de menor desarrollo ^15^.

### Etiología de la hiperplasia condilar

La etiología sobre el desarrollo de los cóndilos mandibulares en HC se debe a múltiples factores que pueden ser hormonales, funcionales, relacionados con el tumor, infecciones recurrentes, traumatismos, neoplasias o la propia genética del individuo ^18^^,^^27^.

### Etiología de la microsomía hemifacial

La causa de la MHF permanece desconocida, pero parece ser un defecto en el desarrollo del primer y segundo arco faríngeo durante las primeras 6 semanas de gestación ^19^.

### Evaluación y diagnóstico de las asimetrías faciales

Se puede evaluar desde varios ángulos el patrón de afuera hacia adentro, en el que la evaluación comienza desde afuera y continúa hasta la dentición intraoral. Se deben evaluar el tejido, la estructura esquelética y la coordinación del arco dental, así como su causa ^11^.

La valoración radiográfica de los parámetros cefalométricos en asimetría esqueletal incluye la evaluación del ángulo SNA, el ANB, el ángulo gonial obtuso, la protrusión mandibular, la relación incisiva y el plano de Frankfort ^26^.

Respecto de la asimetría mandibular, es preciso realizar un diagnóstico diferencial con otras causas de desviación mandibular lateral, como la hiperplasia condilar, la microsomía hemifacial y la tortícolis muscular congénita (Tabla 4) ^17^.

**Tabla 4:** Diagnóstico diferencial entre la asimetría mandibular y otras patologías que causan desviación mandibular

Causa	Anormalidad	Hallazgos diagnósticos
Tortícolis muscular	Deformación de la base craneal	Inclinación de la cabeza y rotación restringida del cuello
Hiperplasia condilar	Anormalidad condilar	Panorámica: altura vertical incrementada del cuello
Microsomía hemifacial	Anormalidad condilar	Hallazgos variables, canteo oclusal Panorámica: cóndilo/rama ausente o hipoplásico. Tejidos blandos: microtia (poco desarrollo del pabellón auricular), macrostomia (hendidura orofacial entre el maxilar superior e inferior)

Fuente: Cueto *et al*., 2015

Existen otros métodos disponibles para el diagnóstico complementario y el análisis más preciso sobre el tipo de asimetría que se presenta, basados en imágenes 3D, lo que permite medir la asimetría facial, al capturar y cuantificar la morfología de la superficie craneofacial. Estos incluyen antropometría directa y fotografía digital, así como nuevos sistemas de imágenes de superficie tridimensionales (3D) ^5^.

La tomografía computarizada 3D (3D-CT) permite visualizar imágenes 3D de tejidos duros y blandos al mismo tiempo, con menos aumento y problemas de distorsión ^10^.

### Tomografía computarizada 3D como método de diagnóstico

Las técnicas de imagenología más nuevas, como la tomografía computarizada tridimensional, se están utilizando para localizar con precisión la discrepancia. Es necesario un estudio detallado de varios registros de diagnóstico obtenidos en el paciente para determinar la causa, la extensión y la ubicación de la asimetría. Esto permitirá al médico formular un plan de tratamiento adecuado ^28^.

La tomografía computarizada de haz cónico (CBCT) es un método preciso y confiable para evaluar las estructuras craneofaciales, pues proporciona una reconstrucción tridimensional (3D) de estructuras anatómicas con alta resolución y sin aumento ^23^. Los datos de la tomografía computarizada se pueden usar para evaluar medidas lineales y angulares después de separar una parte necesaria, como el maxilar o la mandíbula ^29^.

### Tratamiento de las asimetrías faciales

En algunos casos, la asimetría facial viene acompañada por disfunciones más o menos graves, como la alteración de la alimentación o los trastornos respiratorios, que crean problemas adicionales y requieren un tratamiento multidisciplinar. 

Independientemente de la etiología, los problemas asociados con la restauración del correcto funcionamiento y apariencia del rostro son, a menudo, extremadamente difíciles debido a la frecuente coexistencia de anomalías en los tejidos óseos, dentales y blandos ^30^.

El procedimiento clínico en este tipo de patologías comprende tres etapas de trabajo que deben ser correctamente aplicadas para lograr los resultados esperados por el paciente: ortodoncia prequirúrgica, etapa quirúrgica y ortodoncia posquirúrgica. Este trabajo en equipo apunta a optimizar los resultados, lo que reduce las complicaciones a un mínimo manejable y sin secuelas ^31^^,^^32^.

### Manejo ortodóncico

Las asimetrías dentales sin peralte oclusal pueden tratarse mediante un tratamiento de ortodoncia solo a través de mecánicas de extracción asimétricas y una combinación de elásticos intraorales ^28^^,^^33^.

Los tratamientos ortopédicos presentan gran eficacia en niños con maloclusión de clase III a corto plazo. Se utilizan varios aparatos para el tratamiento temprano de la clase III esquelética, incluidos Bionator, Frankel (FR-III), mentonera, aparato de placa doble, aparato de Eschler (aparato progénico) y mascarilla de prolongación ^26^.

### Ortodoncia prequirúrgica

Para el tratamiento de ortodoncia prequirúrgico en pacientes con asimetría facial, se deben tomar en cuenta pautas como las siguientes:

• Las líneas medias dentales no deben coincidir, sino ubicarse en la línea media de cada mandíbula.
• Siempre que se corrija la asimetría facial mediante cirugía de una sola mandíbula, la línea media dental de la mandíbula no operada debe coincidir con la línea media facial.
• Siempre que se cree una mordida abierta unilateral, después de aumentar la altura de la rama en casos de microsomía hemifacial y anquilosis de la articulación temporomandibular, la altura debe mantenerse mientras se permite el crecimiento alveolar vertical del maxilar.
• La descompensación mínima de los incisivos justifica la extracción del segundo premolar superior, mientras que la extracción del primer premolar superior está indicada en aquellos casos en que los incisivos superiores requieren mayores grados de descompensación.
• Se prefiere el abordaje sin extracciones con reaproximación en casos de apiñamiento mínimo que no requieren descompensación dentoalveolar ^34^.

### Manejo ortognático

Aproximadamente el 4% de la población tiene una deformidad dentofacial que requiere tratamiento ortodóncico quirúrgico para corregirla. Las indicaciones más comunes para el tratamiento quirúrgico son las clases II y III esqueléticas severas, y las discrepancias esqueléticas verticales en pacientes que ya no están en crecimiento ^35^. El objetivo del tratamiento no es solo mejorar la estética facial y la oclusión funcional, sino lograr la recuperación del sistema estomatognático ^36^.

La asimetría posicional de la mandíbula debe abordarse antes de que se pueda diagnosticar y corregir eficazmente la asimetría de tamaño y forma, y el único procedimiento para centrar la mandíbula en pacientes adultos es la cirugía ortognática (OGS) ^37^.

### Enfoque de la cirugía primero

Es una técnica novedosa, que no requiere ortodoncia prequirúrgica y con la que se llega a acortar de 1 a 1,5 años el tiempo de tratamiento. Se cree que la dirección del movimiento de los dientes de ortodoncia después de la cirugía se coordina con las fuerzas musculares que aceleran la alineación y la descompensación de los dientes. Los pacientes con apiñamiento leve a moderado y una coordinación aceptable del arco pueden ser los indicados para someterse a una cirugía ortognática sin tratamiento de ortodoncia preoperatorio ^38^.

### Osteotomía SSRO y osteotomía IVRO

La osteotomía sagital de la rama dividida (SSRO) y la osteotomía vertical intraoral de la rama (IVRO) son procedimientos mandibulares ortognáticos que se utilizan de forma rutinaria para la corrección de deformidades dentofaciales asimétricas.

El SSRO, generalmente, se emplea para casos que tienen una magnitud de asimetría leve a moderada (hasta 7-8 mm). Por lo general, se prefiere IVRO para la corrección de asimetrías más grandes (magnitud superior a 8 mm) con síntomas de trastorno temporomandibular. IVRO está indicado para el lado que se mueve en dirección posterior ^34^.

### Cirugía de mandíbula única

La cirugía ortognática aislada se realiza, generalmente, en casos de asimetrías no complicadas que afectan solo la mandíbula, como el prognatismo desviado. El movimiento rotacional transversal asimétrico de los segmentos distales mandibulares se logra mediante osteotomías ramales bilaterales ^34^.

### Cirugía bimaxilar (mandíbula doble)

Para mejorar el estado actual y obtener una oclusión deseable, a menudo, se realiza una cirugía de dos mandíbulas prestando especial atención a la recaída debida a la deficiencia del contacto óseo en la línea de osteotomía y a los cambios de configuración muscular, resultantes del movimiento rotacional del segmento osteotomizado en los planos coronal y axial ^39^.

### Condilectomía alta

En la condilectomía alta, se remueve quirúrgicamente la porción articular condílea donde se encuentra el cartílago en crecimiento o con una condilectomía baja o proporcional, mediante la cual se intenta normalizar el tamaño total de la rama mandibular para corregir la asimetría ^16^.

## DISCUSIÓN

Las asimetrías faciales necesitan un diagnóstico preciso para lograr la mejor planificación del posible tratamiento. La tomografía computarizada es la más utilizada para evaluar las distintas asimetrías, por lo cual algunos autores estudiaron este tipo de análisis y obtuvieron los resultados esperados. Choi ^11^ evaluó la asimetría fluctuante a través de fotogrametría y radiometría bidimensionales y tridimensionales (3D). Ryu *et al*. ^20^ evaluaron los patrones de rotación de las estructuras dentofaciales según diferentes patrones esqueléticos verticales y analizaron su influencia en la desviación del mentón en la deformidad esquelética de clase III con asimetría mandibular. Kamata *et al*. ^10^ determinaron los factores de la asimetría facial en pacientes con prognatismo mandibular, mientras que Lonic *et al*. ^3^ realizaron una investigación a fondo sobre la detección de asimetrías faciales y el peralte oclusal, a través de planos de referencia tridimensional.

En cuanto a la elección del tratamiento, este se ve influenciado por el tipo de asimetría, aunque para la mayoría de estas el tratamiento ortognático es el ideal. Minte *et al*. ^24^ realizaron este procedimiento en una paciente que padecía hiperplasia condilar con asimetría facial, desviación mandibular, desviación de la línea media inferior, mordida abierta y cruzada, y lograron mejorar la estética facial y conseguir una oclusión aceptable. Almeida ^40^ trató a una paciente con asimetría por maloclusión clase III con perfil casi recto y mordida cruzada anterior, y logró corregir la asimetría y obtener un perfil facial más agradable.

## CONCLUSIONES

La asimetría facial se puede describir como una alteración o desarmonía, o en casos más severos como una deformidad de las características faciales de la persona, la cual llega a repercutir en la autoestima, la estética facial y el funcionamiento de todas las estructuras involucradas. Por lo tanto, tiene gran importancia en el área odontológica, al no presentarse una buena aceptación del paciente ante la forma de su cara debido a esta alteración, así como la posible afección de esta sobre la comodidad y el funcionamiento masticatorio.

La asimetría facial puede presentarse de diferentes maneras, como lo son la asimetría dentofacial, bucodental y mandibular, o puede ser una asimetría característica presente en algunos de los factores ya mencionados, por ejemplo, por maloclusión (clase III), microsomía hemifacial o hiperplasia condilar.

De acuerdo con los tratamientos realizados durante los últimos años dentro de la clínica dental, queda comprobado que el tratamiento ortognático acompañado de una planificación previa es la mejor opción para el manejo de las asimetrías faciales esqueletales, pues tiene como resultado el mejoramiento estético y funcional, así como la buena estabilidad, satisfacción y salud posoperatoria, lo que a su vez cumple el propósito de mejorar la morfología de la mandíbula y corregir la relación interoclusal. Sin embargo, en casos de asimetrías más leves o funcionales, pueden llegar a ser corregidas con un simple tratamiento de ortodoncia, con o sin necesidad de extracciones dentales.

## Fuente de financiamiento:

Autofinanciado
